# Patient Management in Aortic Stenosis: Towards Precision Medicine through Protein Analysis, Imaging and Diagnostic Tests

**DOI:** 10.3390/jcm9082421

**Published:** 2020-07-28

**Authors:** Laura Mourino-Alvarez, Tatiana Martin-Rojas, Cecilia Corros-Vicente, Nerea Corbacho-Alonso, Luis R. Padial, Jorge Solis, María G. Barderas

**Affiliations:** 1 Department of Vascular Physiopathology, Hospital Nacional de Paraplejicos (HNP), SESCAM, 45071 Toledo, Spain; lmourino@sescam.jccm.es (L.M.-A.); tatiana_martin_rojas@hotmail.com (T.M.-R.); ncorbacho@sescam.jccm.es (N.C.-A.); lrodriguez@sescam.org (L.R.P.); 2 Hospital Universitario 12 de Octubre and Instituto de Investigación Sanitaria Hospital 12 de Octubre (imas12), 28041 Madrid, Spain; ceciliacorros@yahoo.com (C.C.-V.); jorge.solis@salud.madrid.org (J.S.); 3 Unidad de Imagen Cardiovascular Analiza, 28008 Madrid, Spain; 4 Hospital Virgen de la Salud, SESCAM, 45071 Toledo, Spain; 5 Centro de Investigación Biomédica en Red de Enfermedades Cardiovasculares (CIBERCV), Instituto de Salud Carlos III, 28029 Madrid, Spain

**Keywords:** aortic stenosis, aortic valve, proteomics, biomarkers, precision medicine

## Abstract

Aortic stenosis is the most frequent valvular disease in developed countries. It progresses from mild fibrocalcific leaflet changes to a more severe leaflet calcification at the end stages of the disease. Unfortunately, symptoms of aortic stenosis are unspecific and only appear when it is too late, complicating patients’ management. The global impact of aortic stenosis is increasing due to the growing elderly population. The disease supposes a great challenge because of the multiple comorbidities of these patients. Nowadays, the only effective treatment is valve replacement, which has a high cost in both social and economic terms. For that reason, it is crucial to find potential diagnostic, prognostic and therapeutic indicators that could help us to detect this disease in its earliest stages. In this article, we comprehensively review several key observations and translational studies related to protein markers that are promising for being implemented in the clinical field as well as a discussion about the role of precision medicine in aortic stenosis.

## 1. Introduction

Aortic stenosis (AS) is defined as a narrowing of the aortic valve, leading to the obstruction of blood flow from the left ventricle (LV) to the aorta. AS is a chronic heart disease and the most common valvular disease in developed countries, often leading to aortic valve replacement (AVR) [1]. The prevalence of aortic valve sclerosis, the precursor to AS, increases significantly with age [2]; in fact, about 25% of all 65-year-olds show aortic valve sclerosis, a thickening of the valve without hemodynamic changes [3], and 4–5% have AS [4]. The incidence of this valvulopathy is ever increasing with the aging population.

For a long time, AS has been thought to be a passive degenerative disease, but currently it is known to be an active, complex, and highly regulated pathobiological process involving a multitude of events, such as chronic inflammation, lipoprotein deposition, activation of the renin–angiotensin system, osteoblast differentiation of valvular interstitial cells, and active calcification [5,6].

The discoveries of an association between degenerative AS and traditional atherogenic risk factors such as hypertension, smoking, diabetes, cholesterol levels, and lipoprotein(a) have led to the hypothesis that AS might represent an atherosclerotic-like process [7], raising the possibility that it is preventable. Nevertheless, there are important dissimilarities between AS and atherosclerosis, which suggests a more complex scenario. For example, the implication has been shown of several specific cell-signaling pathways, such as receptor activator of nuclear-κB (RANK) and Runt-related transcription factor 2 (Runx2/Cbfa1), in valvular calcification and AS. In addition, genetic factors may also be taken into account as genetic polymorphisms and mutations have been associated with AS, including a specific vitamin D receptor allele, which has been correlated with increased risk of AS, neurogenic locus notch homolog protein 1(NOTCH1) mutations in bicuspid aortic valve disease [8]. It is clear that the pathophysiology of this disease is complex and multifactorial and involves more than simple valve degeneration.

Here, we review several key observations and translational studies related to the molecular mechanisms of AS development, with a particular focus on the application of precision medicine through new predictive biomarkers based on proteomics with potential clinical significance. We discuss the application of proteomic findings to assign new markers for the early diagnosis of AS, to improve patient management and avoid needless surgical intervention. Finally, we consider the mechanisms and pathways implicated for better-targeted treatment, and we highlight the important research efforts to identify biomarkers in AS disease by different techniques, as well as the difficulties found along the way.

## 2. Problems in Patient Management

Patients with AS may exhibit symptoms such as angina, syncope, or heart failure; however, as many as 50% of patients with severe AS are asymptomatic at the time of diagnosis [9,10,11]. These patients are diagnosed thanks to the detection of a systolic murmur after auscultation or abnormalities in a transthoracic echocardiography indicated for other reasons. Unfortunately, classic physical examination findings are specific but not sensitive for evaluation of stenosis severity. Although a loud late-peaking systolic murmur that radiates to the carotid arteries, a single or paradoxically split-second heart sound, and a delayed and diminished carotid upstroke confirm the presence of severe AS, these indicators may be normal in elderly patients because of the effects of aging on the vasculature.

Nowadays, echocardiogram is the cornerstone of the diagnosis of AS. This technique allows the accurate identification of the aortic valve anatomy and function, specifically valve motion quality. Moreover, it provides valuable prognostic information by assessing valve calcification, left ventricular wall thickness and function, and the presence of other associated valve disease or aortic pathology.

The natural histories of patients with unoperated AS have shown a poor prognosis once a peak aortic valve velocity of >4 m/s is reached, which is equivalent to a mean aortic valve gradient >40 mm Hg. However, severe AS can be present with lower aortic valve velocities and gradient in patients with lower aortic valve gradients.

Transvalvular velocity, mean pressure gradient, and aortic valve area by continuity equation (AVA) are the hemodynamic parameters used to assess AS severity, and all of them can be measured by Doppler echocardiography in almost all patients. Valve area is the ideal measurement for assessing the severity of AS, although it has some limitations in clinical practice and has always to be considered together with flow rate, mean pressure gradient (the most robust measurement), ventricular function, size, and wall thickness. The degree of valve calcification, blood pressure, and functional status must also be considered [12].

As mentioned previously, as many as 50% of patients with severe AS are asymptomatic at diagnosis [9,10,11], and the optimal timing of intervention for these patients is under discussion because it has not been properly defined. Although patients with asymptomatic severe AS have a better prognosis than symptomatic peers [13], in 5 years, approximately 66% will progress, and 75% will have either died or underwent AVR [10]. The follow-up of these patients is critical since the progression of the disease could be highly variable and unpredictable, and the risk of sudden death is 1–1.5% per year. While echocardiography has a central protagonist in the risk stratification of patients with AS, other complementary tests can also be important to guide the optimal timing of AVR, such as treadmill exercise testing, echocardiogram exercise testing, biomarker assessment, and imaging.

On the other hand, patients with symptomatic severe AS and no treatment have an extremely poor prognosis [14,15,16]: up to one-half of patients will die within 1 or 2 years of diagnosis [13,17,18]. Nowadays, AVR-either surgical or transcatheter-is the only effective treatment to improve survival [19,20,21,22,23]. Current American and European guidelines recommend surgical AVR for appropriate patients with severe symptomatic AS by way of its benefit greatly outweighs the risk (class I indication). For transcatheter AVR, this kind of indication (class I) is mainly prescribed for severe symptomatic AS patients who are not candidates for surgical AVR [12,24]. In other cases, the decision between surgical or transcatheter intervention should be made according to individual patient characteristics.

## 3. Protein Markers for Improving Patient Management: Role of Proteomics

Detailed characterization of the proteome, the whole-protein complement of the genome, is the major goal of proteomics that, in the context of disease mechanisms, explores the complexity of proteins in the body fluids and tissues of patients. These proteins may have potential as disease biomarkers, which are well defined as measurable indicators of physiological or pathologic processes or responses to behavioral or therapeutic interventions [25]. Multiple efforts have been made to define useful biomarkers that can classify patients and important advances have been made through the development of diagnostic and prognostic assays for patients presenting with, or at risk of, a multitude of diseases such as cancer [26] or kidney diseases [27].

By their nature, blood and urine are the most widely investigated samples in biomarker research as they are easy to obtain and routinely used in the clinical field. In this case, biomarkers may not only be valuable as diagnostic or prognostic tools, but also for the staging of disease and prediction of clinical response to an intervention [28]. In relation to the assessment of cardiovascular diseases, the use of protein biomarkers has been particularly beneficial, with the notable inclusion of natriuretic peptides and troponin isoforms in clinical decision making for heart failure [29] and acute coronary syndromes [30], respectively. Unfortunately, research on biomarkers for AS is much less extensive, and biomarkers are still needed for stratifying risk and timing of intervention [31]. This is especially important because surgery should be only performed when the risks of the AS outweigh those of interventions. A significant issue is that the assessment of risk is challenging, as clinical guidelines are often based on observational data rather than high-quality randomized controlled trials. Early interventions may expose the patient to an unnecessary risk of complications, including living with a prosthetic valve or lifetime anticoagulation therapy, whereas a delay may produce irreversible damage to the myocardium [32]. Solid tissue samples should also be considered for proteomic analysis, although again this raises ethical issues because it involves sample extraction. An advantage of sampling tissue, however, is that it allows for the direct study of the affected organ, and a quantitative analysis of specific biomarkers in a biopsy could be a critical component in clinical decision-making [33]. In this context, it is important to note that proteomics useful to define is not only diagnostic and prognostic indicators but also therapeutic targets. Tissue samples may thus provide essential information for drug discovery and might allow the correlation of molecular details to histopathological changes found in patient-derived tissues [34]. This molecular knowledge would be essential for discovering new therapies and could be important in diseases such as AS where the only efficient treatment is surgery.

In addition to experimental proteomics, it is essential to highlight the role of computational proteomics. In this sense, the Chou’s five-step rules have been shown to be useful for predicting various attributes of proteins based on their sequence information alone. This workflow is based on the following procedures: (i) construction of benchmark dataset to train and test the model; (ii) representation of the dataset through effective formulation that correlates the biological samples with the target to be predicted; (iii) development of a powerful algorithm to conduct the prediction; (iv) cross-validation tests to evaluate the model; and (v) development of a publicly available and user-friendly web-server for the predictor [35]. Using this approach, remarkable progress has been achieved in predicting protein subcellular location [36] and post-translational modification (PTM) site [37,38]. In biomedical research, this kind of modeling is becoming more common. For example, in multiple drugs testing, it allows the selection of the more promising combinations and drastically reduces the economic costs and time consumption [39,40].

Finally, it is important not to forget that there are still huge challenges in the management of patients by proteomics techniques. The translation of the verified biomarkers for prognosis, diagnosis, risk stratification, and treatment in the daily clinical practice involves a very important commitment between clinicians and researchers in order to establish these potential biomarkers into the hospital’s core lab testing [41]. Once all proteins candidates to be potential biomarkers of disease have been tested in large cohorts of patients in order to define useful clinical panels of disease, they require standardization for being properly implemented as routine tests. At this point, targeted proteomic approaches by means of mass spectrometry, such as selected reaction monitoring, will be perfectly suitable for analyzing large populations. One of the most important issues is developing appropriate methods and evaluating data quality between different laboratories as using this method it is more difficult to compare the results between hospitals. This is much more simple when using commercial immunoassay reagents. Another drawback of targeted proteomics is the initial high cost of the equipment. Nevertheless, the cost of the reagents used to run the instrument as well as the cost of the extraction are usually lower than that of the immunoassay reagents that are usually used in these laboratories [42]. In any case, it is worth trying to solve all these small inconveniences to take advantage of the high sensitivity and specificity of this methodology.

### 3.1. Classical Approach for Evaluating Disease: The Use of Individual Markers

Lately, many potential biomarkers or candidates have been evaluated to diagnose AS and monitor its progression and prognosis. Given the nature of the disease, biomarkers belonging to multiple biological pathways such as endothelial dysfunction, oxidative stress, inflammation, or mineral, and lipid metabolism have been studied, although only a few have proved promising [31,43]. For example, proteins involved in bone formation, such as fetuin-A, osteopontin, and osteoprotegerin, have a strong association with AS [44,45,46], but it is not clear whether or not they are related to disease progression. Indeed, in a study of 88 patients with different degrees of calcification and concomitant coronary artery disease, fetuin-A levels were not significantly different between patients with no AS, moderate AS, ands severe AS [47]. Nonetheless, Feistritzer et al. have demonstrated the relation of low plasma fetuin-A levels with a prolonged inflammatory response in left ventricular remodeling [48]. Thus, the chronic inflammatory process that involves AS could be aggravated by the low levels of fetuin-A found in AS patients. A similar situation is seen with low-density lipoproteins (LDLs). While their relationship with AS is well established [49,50], their usefulness to predict progression of AS is unclear. Mohty et al. (2008) examined 102 explanted AS valves, ranging from mild fibrosis thickening to severe thickening and calcification. They found differences in oxidized LDL and small LDL particles, but the comparison of the plasma lipid profile revealed no significant difference between groups for total cholesterol, LDL-cholesterol, and high-density lipoprotein (HDL)-cholesterol [51]. Moreover, several major studies (Simvastatin and Ezetimibe in Aortic Stenosis -SEAS-, Scottish Aortic Stenosis and Lipid Lowering Trial, Impact on Regression -SALTIRE-, and Aortic Stenosis Progression Observation: Measuring Effects of Rosuvastatin -ASTRONOMER-) failed to prove the efficacy of LDL-lowering therapy in retarding the progression of [52,53,54]. By contrast, high levels of lipoprotein(a) are directly linked to AS [7] and are also associated with faster progression of the disease and increased risk of AVR, as demonstrated in two independent studies with 454 [55] and 220 [56] patients diagnosed with AS. These findings support the hypothesis that lipoprotein(a) mediates AS progression and provides a rationale for randomized trials of lipoprotein(a)-lowering therapies in AS. C-reactive protein, an inflammatory marker, is a well-recognized predictor of cardiovascular events [57,58,59], but again, it is not useful for predicting progression from sclerosis to stenosis [60], as demonstrated in a large, randomly selected, population-based cohort of 5621 participants where approximately 9% of subjects with aortic sclerosis progressed to AS over a 5-year follow-up period. After multivariable analysis, the authors concluded that C-reactive protein appears to be a poor predictor of subclinical calcific aortic-valve disease.

Other markers implicated in myocardial stress or cardiac remodeling, which are direct consequences of AS, have also been studied for their suitability as biomarkers. For example, B-type natriuretic peptide (BNP) and its prohormone, N-terminal pro-brain natriuretic peptide (NT-proBNP), have been proposed as useful markers for risk stratification and predictors of prognosis in patients with asymptomatic AS [61]. Likewise, Monin et al. (2009) defined a score based on peak aortic-jet velocity, BNP level, and female sex to predict outcomes in individual patients with asymptomatic AS [62]. After a prospective study of 237 patients with moderate/severe AS, Farré et al. (2014) concluded that NT-proBNP determination may be used to adapt the Monin score [63]. Specifically, high BNP or NT-proBNP levels were related to lower survival and higher probability of developing symptoms.

Troponins also seem to be promising biomarkers for use in managing patients with AS as they are associated with long-term risk of AVR and mortality, especially high-sensitivity cardiac troponin T [64] and high-sensitivity cardiac troponin I (cTnI) [65]. Moreover, the combination of high-sensitive cardiac troponin T and NT-proBNP has been identified as a significant predictor of all-cause mortality in multivariable analysis [66].

Although some of these individual markers are promising, biomarkers in general have not played a significant role in the evaluation and management of patients with AS, with the exception of troponins and BNP [67]. Besides, many of them belong to common processes such as inflammation, and so they are not specific for AS and may lead to errors in diagnosis.

### 3.2. Proteomics for Defining Biomarker Panels

Ideally, assessment of global risk likely requires the integration of multiple biomarkers (including clinical factors) and the evaluation of biomarkers belonging to independent pathways. This issue has been demonstrated in heart failure populations without AS [68,69]. For this purpose, -omics tools are powerful, as they allow the definition of panels of biomarkers that may be later assessed in numerous cohorts of patients. Protein biomarkers are of special interest to the clinical field due to the availability of a large range of analytical instrumentation that can identify and quantify proteins in complex biological samples.

**Figure 1:**
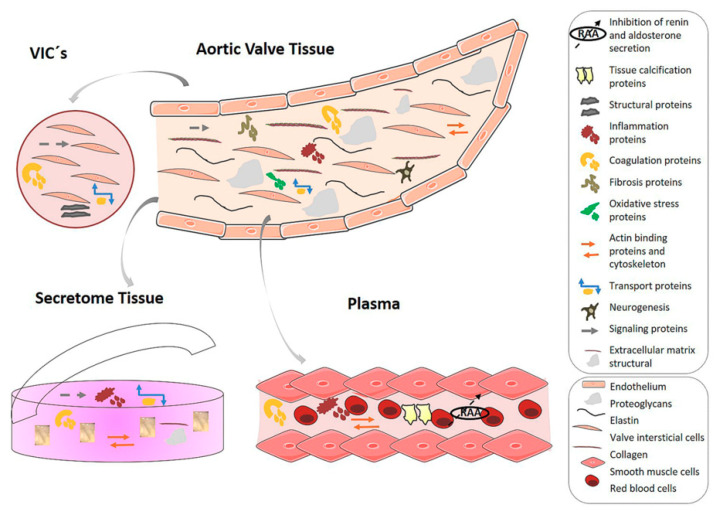
Processes involved in aortic stenosis development. Mechanisms involved in the pathogenesis of aortic stenosis and functional implications of observed protein alterations related to tissue calcification, structural, inflammation, coagulation, fibrosis, oxidative stress, actin binding and cytoskeleton, transport, neurogenesis, signaling, extracellular matrix modulation, and inhibition of renin and aldosterone secretion. The figure shows the four types of samples described in this review: valvular interstitial cells (VICs) and aortic valve tissue (on the top); secretome and plasma (on the bottom).

Several proteomics studies have been performed using different sample types to unravel the molecular mechanisms of AS, provide potential indicators at early stages of the disease, and identify new therapeutic targets (Figure 1). For the latter purpose, cell or tissue analyses are likely the most suitable platforms as they are ultimately the targets for new drugs and should provide important information for treatment discovery. As a model for valve-cell calcification, the proteome of bovine aortic interstitial valve cells was studied after endotoxin treatment using two-dimensional electrophoresis followed by mass spectrometry [70]. The authors found 34 unique cytosolic and 10 unique membrane-associated proteins that were significantly altered after the pro-calcifying treatment. Analysis showed that the proteins were involved in different cellular functions, including chaperone-mediated protein folding, protein metabolism and transport, cell redox/nitric oxide homeostasis, and cytoskeletal organization. In a similar experimental procedure, Yu et al. (2018) studied the effect of lipoprotein(a) on human aortic valve interstitial cells using mass spectroscopy to identify the different lipoproteins and oxidized phospholipids in calcified aortic valves [71]. The results showed that cells treated with either LDL or lipoprotein(a) had significantly higher calcium deposition compared with control cells, pointing to lipoprotein(a) as a potential therapeutic target to manage the progression of AS. It is important to note here that proteomic analysis of calcified tissue is challenging; however, an effective protein extraction protocol for the proteome analysis of AS valves was described several years ago [72]. This protocol has been used to analyze aortic valve tissue by two-dimensional fluorescence difference gel electrophoresis (2D-DIGE), which revealed differences in the abundance of 17 proteins, 12 up-regulated in calcified tissue and 5 down-regulated [73]. These proteins were involved in different pathways such as fibrosis, coagulation, and inflammation. In a later analysis, proteins related to inflammation, oxidative stress, cytoskeleton, and transport were discovered using isobaric tags for relative and absolute quantitation (iTRAQ) methodology, which allowed the identification of a specific protein profile linked to the disease and highlighted the importance of extracellular matrix proteins [74]. Procollagen C-endopeptidase enhancer 1, extracellular superoxide dismutase (Cu-Zn), immunoglobulin gamma-1 chain C region, fibrinogens, and lumican have also been shown to be altered in AS in a study using proteins extracted from thickened and calcified areas and non-diseased areas from the same valve leaflet [75]. The latter study also revealed the importance of post-translational modifications, specifically that low levels of glycosylation of lumican in the thickened and calcified areas of AS aortic valves may be associated with the pathophysiology of AS.

A step forward in the in situ analysis of proteins in tissues has been the development of matrix-assisted laser desorption/ionization-imaging mass spectrometry (MALDI-IMS), a novel proteomic tool that allows the investigation of the physiopathological variations occurring directly in tissue while retaining the histopathological context. Using this methodology, Mourino-Alvarez et al., (2016) were able to describe the spatial distribution of proteins and peptides in aortic valve tissue by analyzing consecutive slices, that is, directly from the surface of the histological sections [76]. Peptide extraction from the tissue followed by liquid chromatography mass spectrometry analysis provided the identification of collagen VI α-3 and NDRG2 (N-myc downstream-regulated gene-2) correlating with the mass obtained by MALDI-IMS and were confirmed by immunohistochemistry.

It should also be considered that in the context of AS, proteins might be secreted from cells and tissues constituting a plasma subproteome named secretome which could provide a direct approximation of the in vivo situation. In the case of aortic valves, a study carried out by Alvarez-Llamas et al. (2013) showed that non-stenotic and stenotic valves cultured in tissue culture medium containing labeled amino acids synthesized and released different proteins and demonstrated the origin of the secreted proteins, ruling out a blood origin [77]. Specifically, they found differences in proteins implicated in extracellular matrix remodeling such as PEDF (pigment epithelium-derived factor), cystatin, and clusterin, highlighting the link between aortic stenosis and atherosclerosis. Based on the premise that these changes may be also reflected in plasma proteome, the same authors extended this analysis to a rabbit model to characterize protein alterations associated with mild calcified valve tissue [78]. The authors first searched for protein differences in valve tissue and, subsequently, looked for these differences in rabbit and human plasma. The results of this analysis allowed them to define a molecular panel comprising three proteins (transitional endoplasmic reticulum ATPase, tropomyosin α-1 chain, and L-lactate dehydrogenase B chain) related to osteoblastic differentiation, which showed a greater discriminative power for calcific aortic tissue than each protein alone.

Analyses of plasma are principally focused on searching for diagnostic and/or prognostic biomarkers. One of the main aspects to consider is that plasma is a biological sample that is easy to obtain and non-invasive for clinical diagnosis. Nevertheless, its complexity and wide dynamic range frequently necessitates a pre-fractionation step to deplete the most abundant proteins (albumin, etc.), allowing the analysis of less abundant proteins. Gil-Dones and colleagues (2012) performed a comprehensive proteomic study using crude, depleted, and equalized plasma to address this question and, at the same time, to describe plasma differences between healthy controls and patients with AS [79]. They found that the three individual strategies employed (non-fractionated and immunoaffinity-fractionated plasma for abundant plasma proteins and “equalized” plasma by peptide saturation) were complementary and had a consistent level of concordance. In line with the results from tissue and secretome studies, they found that AS is an active and inflammatory process with important alterations in blood homeostasis and coagulation, impairing plasma proteases and protease inhibitors. While these studies provide essential information about AS mechanisms, information is lacking on the sensitivity and specificity of the indicators. In 2016, Mourino-Alvarez and colleagues combined metabolomics and proteomics to define four molecular panels in patients with calcific AS according to different biological functions: coagulation, inflammation and immune response, response to ischemia, and lipid metabolism [78]. Receiver operating curves of these panels were used to determine sensitivity and specificity, showing *p*-values lower than 0.05 in all cases. The use of multiple biomarkers has also been proven in a cohort of 345 patients with severe AS undergoing AVR [80], demonstrating the potential utility of multiple biomarkers that reflect diverse biological pathways (cardiac and systemic) to improve risk stratification of patients with AS. Specifically, the authors found that an increased number of elevated biomarkers (GDF15 (growth differentiation factor 15), sST2 (soluble suppression of tumorigenesis-2), and NT-proBNP) was associated with a higher risk of mortality after ARV. Although these findings are promising, further studies are needed to evaluate how such biomarker panels might influence patient management and treatment decisions and to assess an optimal cut-off point of the different indicators to guide decision-making in clinical practice. For that purpose, larger cohorts including different kind of patients (asymptomatic, symptomatic, with several risk factors) should be evaluated in controlled clinical trials.

## 4. Future Clinical Perspectives: The Importance of Precision Medicine in AS

The goals of clinical practice, both in AS and in other diseases, have always been directed to the patients, to provide relief from pain, to cure the disease, and to promote health to prevent illness from occurring. Classically, reductionism in medicine presupposes that patients with common signs and symptoms share the same disease pathophenotype and will respond similarly to medical interventions tested in groups of similar subjects [81].

**Figure 2:**
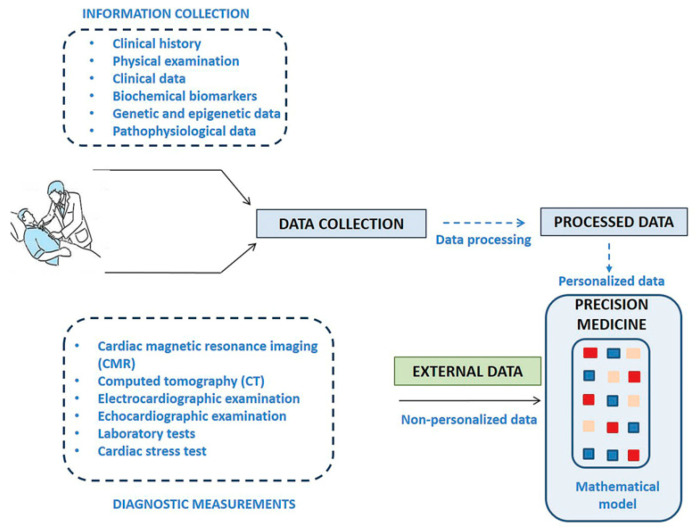
Precision Medicine workflow. The process involves multiple sources of heterogeneous data, including experimental evidence, bioinformatics databases, lifestyle measurements, imaging, and environmental influences, among others. All of them are analyzed through a system integration that incorporates a mathematical model using machine-learning algorithms to identify potential biomarkers and disease networks quickly and accurately, stratify patients and, ultimately, predict more efficacious therapies.

On the contrary, precision medicine is focused on selecting the appropriate treatment for each patient according to their personal pathophenotype. Accordingly, precision medicine combines standard clinical evaluation, including synthesis of data, imaging, or laboratory tests, with different -omics approaches such as genomics, transcriptomics, proteomics, or metabolomics, for deep phenotyping (Figure 2) [82,83].

It is hoped that the application of precision medicine in AS will improve patient management by promoting adequate prevention and treatment options, which should reduce the number of surgeries as well as the economic costs [84,85]. This multimodal approach might be especially useful for decision-making in patients with asymptomatic AS rather than patients with AS. In particular, novel imaging approaches could help to guide the optimal timing of AVR in asymptomatic patients. Indeed, AS is an ideal candidate for precision medicine as it meets several criteria: it is classified according to its symptoms rather than its etiology, its underlying complexity and heterogeneity have not been characterized, and its presentation varies over an extended time frame [86]. In this context, a great effort is being made in developing machine-learning approaches for automated imaging analysis and diagnosis and for predicting outcomes [87]. Recently, Casaclang-Verzosa et al. (2019) published a study where they identified subtypes of AS with high resolution by using aortic valve area, LV ejection fraction, LV mass index, and relative wall thickness [88]. They developed a patient similarity network by means of topological data analysis from cross-sectional echocardiographic data collected from 246 patients with AS. Using this approach, they found a path of AS progression that was also validated in a murine AS model, and they concluded that integrated valvulo-ventricular stratification of subgroups with AS may be a promising approach for predicting the natural progression of the disease.

Unfortunately, much remains to be done in this field. For example, precision medicine could be used to define which patients would benefit from transcatheter instead of surgical aortic valve replacement (SAVR). So far, the majority of patients undergoing transcatheter aortic valve implantation (TAVI) have been considered inoperable or high-risk for conventional SAVR, as recommended by guidelines [89]. The guidelines show that 30-day mortality rates range from 5 to 15% and reported 1-year survival for TAVI ranges from 60 to 80%, largely depending on the severity of comorbidities. Moreover, bleeding in TAVI patients is approximately 31% at 5 years, which increases stroke rates [90]. Nevertheless, there are several trials reporting non-inferiority of TAVI in intermediate-risk patients so this procedure could be considered for the treatment of patients with AS and low to intermediate operative risk, as TAVI is associated with faster recovery and shorter index hospitalization [20,91,92]. Performing different studies considering different comorbidities and analyzing the causes of the fatal outcomes may change clinical recommendations and will be a step forward in precision medicine.

## 5. Conclusions

The management of patients with AS is a challenge for clinicians so the definition of new indicators and preventive strategies is decisive for the implementation of early diagnosis techniques and precision medicine. Risk stratification before symptoms appear in combination with the selection of the most adequate preventive treatment for each patient will allow the reduction of the number of aortic valve replacement and will allow improved survival in AS patients, thus reducing the economic and social burden of this disease.
